# Prophylactic cholecystectomy offers best outcomes following ERCP clearance of common bile duct stones: a meta-analysis

**DOI:** 10.1007/s00068-022-02070-2

**Published:** 2022-09-02

**Authors:** Gearóid Mc Geehan, Conor Melly, Niall O’ Connor, Gary Bass, Shahin Mohseni, Magda Bucholc, Alison Johnston, Michael Sugrue

**Affiliations:** 1 Donegal Clinical Research Academy, Letterkenny University Hospital, Donegal, Ireland; 2 School of Medicine, University of Limerick, Limerick, Ireland; 3 Division of Traumatology, Emergency Surgery and Surgical Critical Care, University of Pennsylvania, Philadelphia, USA; 4 Department of Trauma and Emergency Surgery, Orebro University Hospital and School of Medical Sciences, Orebro University, Orebro, Sweden; 5 Department of Surgery, Letterkenny University Hospital, Letterkenny, Co Donegal Ireland; 6 Intelligent Systems Research Centre, School of Computing, Engineering and Intelligent Systems, Ulster University (European Union Interreg VA Funded), Magee Campus, Northern Ireland, UK; 7 EU INTERREG Emergency Surgery Outcome Advancement Project, Centre for Personalised Medicine, Letterkenny, Ireland

**Keywords:** Choledocholithiasis, Cholecystectomy, ERCP, Cholangitis, Biliary outcomes

## Abstract

**Background:**

Symptomatic calculus biliary disease is common with associated morbidity and occasional mortality, further confounded when there is concomitant common bile duct (CBD) stones. Choledocholithiasis and clearance of the duct reduces recurrent cholangitis, but the question is whether after clearance of the CBD if there is a need to perform a cholecystectomy. This meta-analysis evaluated outcomes in patients undergoing ERCP with or without sphincterotomy to determine if cholecystectomy post-ERCP clearance offers optimal outcomes over a wait-and-see approach.

**Methods:**

A Prospero registered meta-analysis of the literature using PRISMA guidelines incorporating articles related to ERCP, choledocholithiasis, cholangitis and cholecystectomy was undertaken for papers published between 1st January 1991 and 31st May 2021. Existing research that demonstrates outcomes of ERCP with no cholecystectomy versus ERCP and cholecystectomy was reviewed to determine the related key events, complications and mortality of leaving the gallbladder in situ and removing it. Odds ratios (OR) were calculated using Review Manager Version 5.4 and meta-analyses performed using OR using fixed-effect (or random-effect) models, depending on the heterogeneity of studies.

**Results:**

13 studies (*n* = 2598), published between 2002 and 2019, were included in this meta-analysis, 6 retrospective, 2 propensity score-matched retrospective studies, 3 prospective studies and 2 randomised control trials from a total of 11 countries. There were 1433 in the no cholecystectomy cohort (55.2%) and 1165 in the prophylactic cholecystectomy (44.8%) cohort. Cholecystectomy resulted in a decreased risk of cholecystitis (OR = 0.15; CI 0.07–0.36; *p* < 0.0001), cholangitis (OR = 0.51; CI 0.26–1.00; *p* = 0.05) and mortality (OR = 0.38; CI 0.16–0.9; *p* = 0.03). In addition, prophylactic cholecystectomy resulted in a significant reduction in biliary events, biliary pain and pancreatitis.

**Conclusions:**

In patients undergoing CBD clearance, consideration should be given to performing prophylactic cholecystectomy to optimise outcomes.

## Introduction

Choledocholithiasis presents as a spectrum of significant clinical challenges including abdominal pain, jaundice, cholangitis and gallstone pancreatitis [1, 2] and is associated with increased long-term mortality. Clearance of the common bile duct is an essential part of the management of symptomatic biliary calculus disease and there is increasing evidence that clearance of the common bile duct may optimise outcomes, particularly in elderly patients [2, 3].

Following Cotton’s pioneering description of cannulation of the common bile duct (CBD) and McCune’s first successful ERCP in the mid-1970’s, ERCP has been widely adopted as the index therapeutic modality for clearance of CBD stones [4–6] and is most commonly performed either prior to or after operative removal of the gallstone reservoir at laparoscopic cholecystectomy. There have been variable approaches to sphincterotomy during ERCP with some preferring balloon dilation and others sphincterotomy [7–9]. Increasingly, ERCP is performed intra-operatively with a rendezvous technique with favourable outcomes reported [10–12]. Primary ERCP is associated with successful clearance of CBD stones in 90%, with a complication rate approaching 10% [13, 14].

Between 10 and 20% of patients with choledocholithiasis have associated cholecystitis with potential resultant progression of cholecystitis. Upward stage migration in disease severity to fulminant or gangrenous cholecystitis is associated with excess morbidity and mortality [2, 15].

Patients who have choledocholithiasis who undergo ERCP have more difficult cholecystectomies with higher conversion rates, morbidity and associated complications [16]. Patients who undergo ERCP alone without cholecystectomy have a 20% readmission rate due to complications relating to the retained gallbladder as a stone reservoir [2].

For this reason we need clarity about the potential benefits and complications of cholecystectomy post-ERCP clearance of CBD stones. Recent meta-analysis, evaluating publications to 2019, suggested that cholecystectomy is preferred [17]. Our meta-analysis evaluated outcomes in all patients undergoing ERCP with or without sphincterotomy to determine if cholecystectomy post-ERCP clearance offers optimal outcomes over a wait-and-see approach.

## Methods

### Search strategy and study eligibility

A systematic review and meta-analysis of the literature was undertaken to incorporate articles relating to ERCP, choledocholithiasis, cholangitis and cholecystectomy. Existing research that demonstrates the outcomes of ERCP with no cholecystectomy versus ERCP and cholecystectomy was reviewed to determine the optimal outcome.

A review of all published English articles was conducted using the PubMed version of Medline, Scopus and Web of Science electronic databases. To assess contemporary evidence, only studies published between 1st January 1991 and 31st May 2021 were included. A literature search was conducted using subject headings, keywords and free text terms for the keywords and their variations. The following medical subject heading (MESH) terms were included: (("cholangiopancreatography, endoscopic retrograde"[MeSH Terms] OR ("cholangiopancreatography"[All Fields] AND "endoscopic"[All Fields] AND "retrograde"[All Fields]) OR "endoscopic retrograde cholangiopancreatography"[All Fields] OR "ercp"[All Fields]) AND ("cholecystectomy"[MeSH Terms] OR "cholecystectomy"[All Fields] OR "cholecystectomies"[All Fields]) AND ("choledocholithiasis"[MeSH Terms] OR "choledocholithiasis"[All Fields] OR ("cholangitis"[MeSH Terms] OR "cholangitis"[All Fields] OR "cholangitides"[All Fields]))) AND ((humans[Filter]) AND (1991/1/1:2021/1/1[pdat]) AND (english[Filter]) AND (alladult[Filter])). These MeSH terms were used to search PubMed and Scopus. The reference sections of reviewed studies were examined for further papers not identified by the initial search strategy. Citations were collated with Microsoft Excel and duplicates removed.

Due to the nature of the current study, no ethical approval was sought.

### Inclusion and exclusion criteria

The methods of the analysis and inclusion criteria were specified in advance to avoid selection bias and documented in a protocol which was registered and published with the PROSPERO database (International Prospective Register of Systematic Reviews, www.crd.york.ac.uk/prospero, registration number: CRD42021257795 on 18th of July 2021). This meta-analysis adhered to the Preferred Reporting Items for Systematic Reviews and Meta- Analyses (PRISMA) statement [18].

To be included, studies had to satisfy the following pre-determined criteria: (1) include ERCP; (2) report post-ERCP complications; (3) design being a randomised controlled trial, prospective observational or retrospective cohort study; (4) reporting ten or more patients in total in sample size; (5) full text articles in the English language.

The search terms used were choledocholithiasis, cholecystectomy, ERCP and cholangitis.

Studies were not included if they (1) were designed as case reports, letters or with < 10 patients; (2) contained paediatric or pregnant populations; (4) did not have comparative cohorts.

### Eligibility assessment and data extraction

We screened titles and abstracts, reviewed full texts and extracted data. Eligibility assessment was performed independently in a blinded standardised manner by three reviewers (GMG, CM, NOC). We resolved disagreements by consensus and if no agreement could be reached a fourth reviewer (AJ) decided.

Three reviewers (GMG, CM, NOC) independently assessed each published study for the quality of study design and risk of bias by using standardised pre-piloted forms and methodological index for non-randomised studies (MINORS) score [19]. A MINORS score of ≥ 16 out of 24 for comparative studies was considered the standard for inclusion.

A standardised data sheet was developed. Information was extracted from each included study on post-ERCP outcomes, study design, country, study length and cohort sizes.

The primary outcome in this study was complication rates following ERCP with no cholecystectomy vs. complications for cholecystectomy post-ERCP. Complications included cholecystitis, cholangitis, all-cause and biliary mortality, pancreatitis, biliary pain and biliary events. A biliary event consisted of (1) recurrent biliary event; (2) cholecystitis; (3) cholangitis; (4) biliary pain; (5) pancreatitis; (6) choledocholithiasis; and (7) biliary malignancy.

### Statistical analysis

For comparison of complication rates of gallbladder in situ and cholecystectomy post-ERCP, odds ratios (OR) were calculated using Review Manager Version 5.4 (Copenhagen: The Nordic Cochrane Centre, The Cochrane Collaboration, 2008). Meta-analyses were performed by computing the OR using fixed-effect (or random-effect) models, depending on the heterogeneity of studies. An OR and Confidence interval (CI) of > 1.0 indicated greater risk of an adverse event occurring in the experimental group.

Heterogeneity was assessed using the *I*^2^ statistic where a value greater than 50% was considered high and a random-effect model was then used to combine variables of interest [20]. OR and 95% confidence intervals (CI) were calculated for each complication following ERCP, along with the *p* value for which a value < 0.05 represented statistical significance.

### Assessment of risk of bias in included studies

**Fig. 1:**
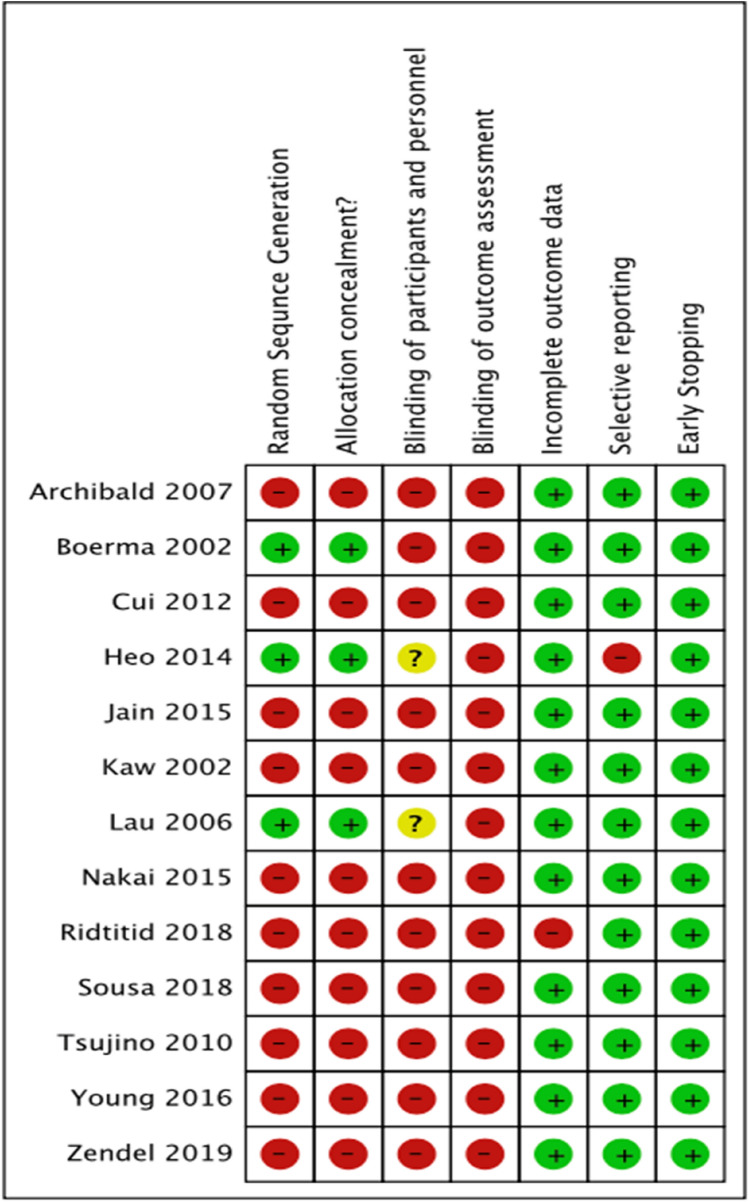
Risk of bias summary: review authors’ judgements about each risk of bias item for each included study

Cochrane ‘Risk of bias’ tool assessed bias, as specified in chapter 8 of the Cochrane Hand-book for Systematic Reviews of Interventions [21], for the following domains; (1) random sequence generation (2), allocation concealment (3), blinding of participants and personnel (4), blinding of outcome assessment (5), incomplete outcome data (6), selective reporting bias (7) and early stopping (Fig. 1).

## Results

**Fig. 2:**
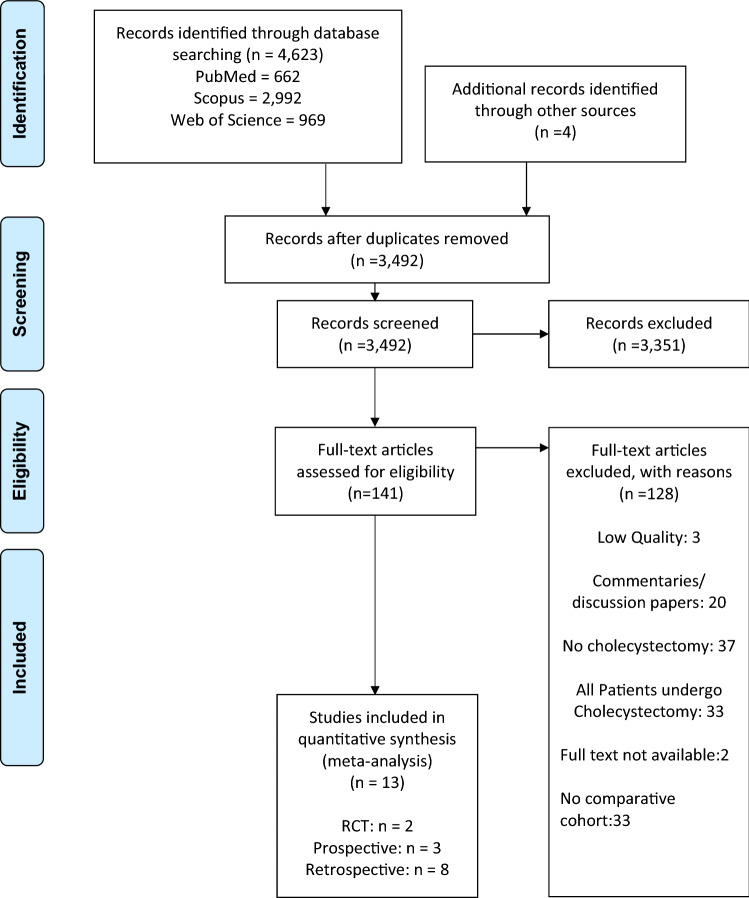
Identification, review and selection of articles included in the meta-analysis of impact of prophylactic cholecystectomy following ERCP

A total of 13 studies (*n* = 2598), published between 2002 and 2019, were included in this meta-analysis. Six retrospective, two propensity score-matched retrospective studies, three prospective studies and two randomised control trial from a total of 11 countries met inclusion criteria (Fig. 2). There were 1433 in the no cholecystectomy cohort (55.2%) and 1165 in the prophylactic cholecystectomy (44.2%) cohort. Baseline characteristics of included studies are shown in Table 1.

**Table 1:** Characteristics of studies used in meta-analysis

Author and year	Country	Study design	Data collection period (years)	Sample size	Age (mean) Cholecystectomy (years)	Age (mean) No cholecystectomy (years)	Female: male	Surveillance (months)	Type of ERCP*
Archibald 2007	Canada	Retro	7	310	50	66	189:121	Not stated	ERCP + ES
Cui 2012	South Korea	Retro	4	232	64	72	112:120	73	ERCP + ES
Jain 2015	England	Retro	2.8	97	85		71:42	41	ERCP
Nakai 2015	Japan	Retro, propensity matched	18	294	66	66	97:197	50	ERCP + BD
Ridtitid 2018	Thailand	Retro	11	79	59	63	47:32	50	ERCP + ES
Sousa 2018	Portugal	Retro	2	131	79	82	75:56	24	ERCP
Young 2016	Taiwan	Retro, propensity matched	12	670	79	79	379:291	Not stated	ERCP + ES
Zendel 2019	Israel	Retro	2	100	73	85	45:55	20	ERCP + EP
Boerma 2002	Switzerland	Pro	4.4	108	60	63	59:49	30	ERCP + ES
Kaw 2002	USA	Pro	4	117	48	58	80:37	33	ERCP + ES
Tsujino 2010	Japan	Pro	14	194	< 60		n/a	67	ERCP + BD
Lau 2006	China	RCT	2.4	178	71	72	86:92	62	ERCP + ES
Heo 2014	South Korea	RCT	2.6	88	64	64	38:50	42	ERCP

*ERCP = endoscopic retrograde cholangiopancreatography

*ERCP + ES = ERCP + endoscopic sphincterotomy

*ERCP + BD = ERCP + balloon dilatation

*ERCP + EP = ERCP + endoscopic papillotomy

### Outcome: cholecystitis

**Fig. 3:**
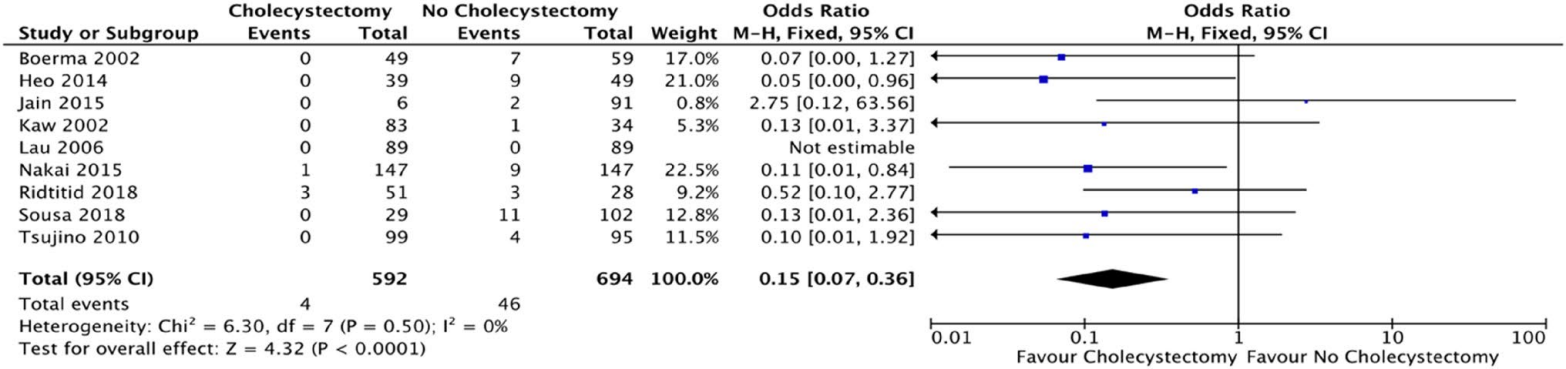
Forest plot: cholecystectomy vs. no cholecystectomy effect on subsequent cholecystitis

Cholecystitis was reported in 9 out of the 13 studies; based on these studies, cholecystectomy compared to no cholecystectomy resulted in a significant decreased risk of cholecystitis (OR = 0.15; CI 0.07–0.36; *p* < 0.0001) (Fig. 3).

### Outcome: cholangitis

**Fig. 4:**
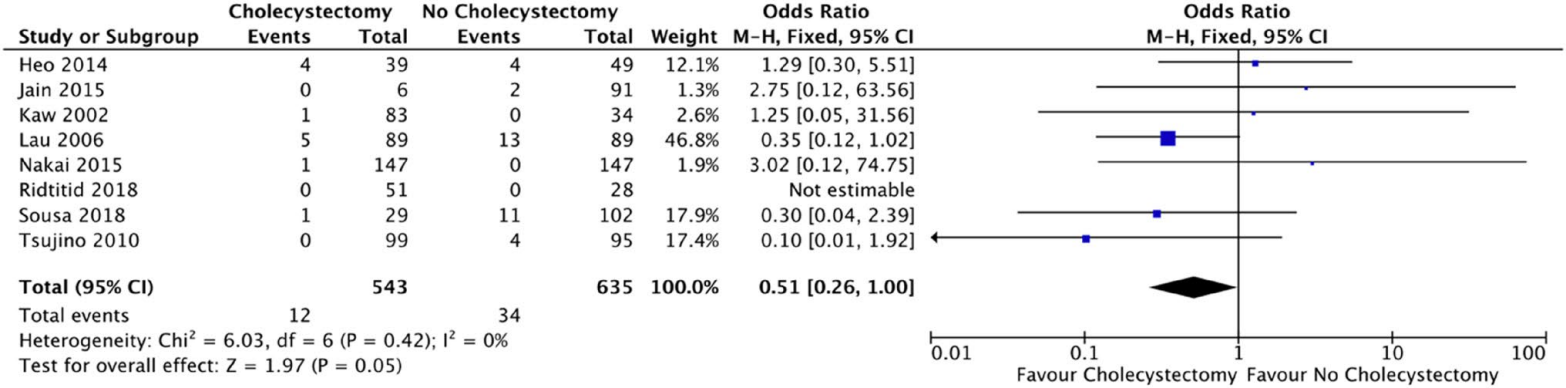
Forest plot: cholecystectomy vs. no cholecystectomy effect on cholangitis

Cholangitis was reported in 8 out of the 13 studies. There was a trend toward decreased risk of cholangitis in patients with cholecystectomy (12/543 [2.2%]) compared to no cholecystectomy (34/635 [5.35%]), (OR = 0.51; CI 0.26–1.00; *p* = 0.05) (Fig. 4).

### Outcome: mortality

**Fig. 5:**
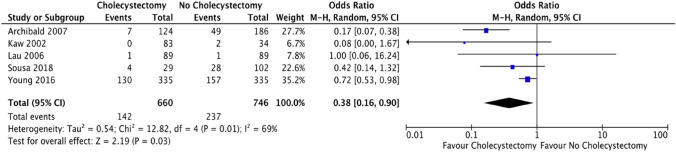
Forest plot: cholecystectomy vs. no cholecystectomy effect on mortality

Five of the included studies compared overall mortality following ERCP, cholecystectomy versus no cholecystectomy with a significant reduction in mortality in those undergoing cholecystectomy (142/660 [21.5%]) compared to no cholecystectomy (237/746 [31.8%] (OR = 0.38; CI 0.16–0.9; *p* = 0.03) (Fig. 5).

### Outcome: biliary pain

**Fig. 6:**
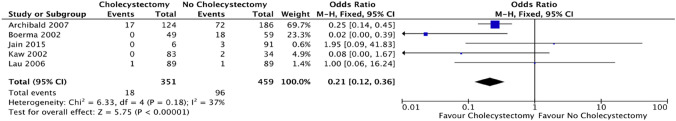
Forest plot: cholecystectomy vs. no cholecystectomy effect on biliary pain

Biliary pain was reported in five included studies showing a significant decrease in biliary pain in the cholecystectomy group (18/351 [5.1%]) in comparison to the no cholecystectomy group (96/459 [20.9%]). (OR = 0.21; CI; 0.12–0.36; p < 0.000001) (Fig. 6).

### Outcome: pancreatitis

**Fig. 7:**
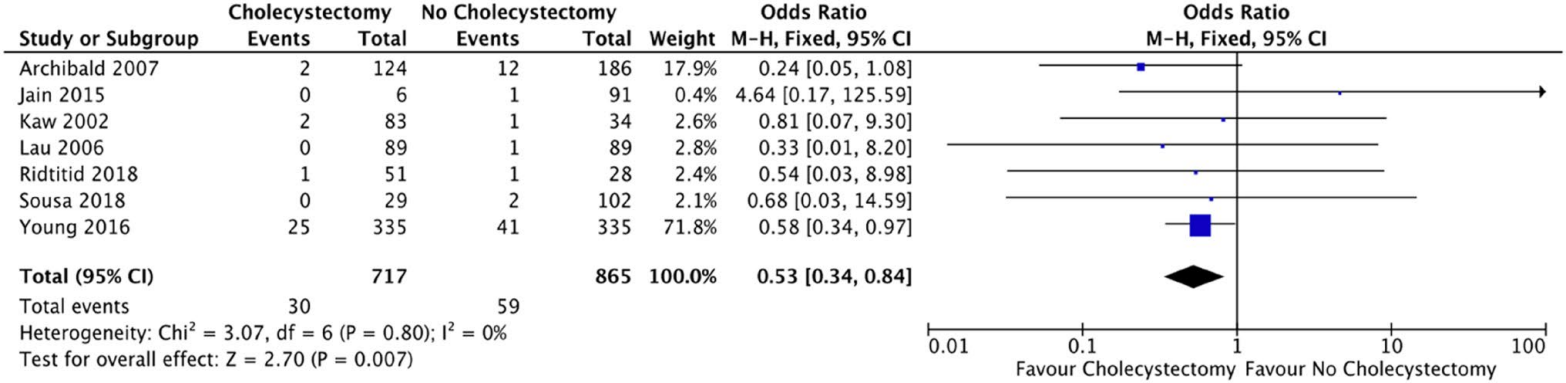
Forest plot: cholecystectomy vs. no cholecystectomy effect on pancreatitis

Pancreatitis was illustrated in 7 out of the 13 included studies. Pancreatitis was shown to have a significant lower risk in the cholecystectomy cohort (30/717 [4.2%]) of 2.6% in comparison to the no cholecystectomy cohort (59/865 [6.8%]) following ERCP. (OR = 0.53; CI 0.34–0.84; *p* = 0.007) (Fig. 7).

### Outcome: biliary events

**Fig. 8:**
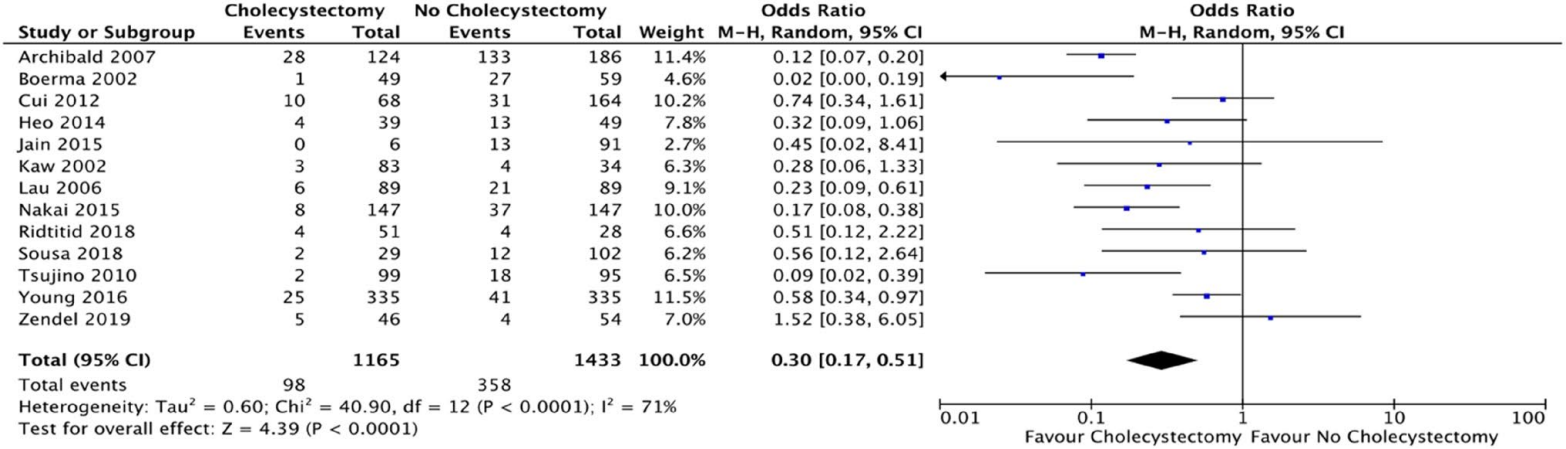
Forest plot: cholecystectomy vs. no cholecystectomy effect on biliary events

Biliary events were reported in all 13 of the included studies. The cholecystectomy group (98/1165 [8.4%]) had a significantly decreased risk by 16.6% of biliary events in comparison to the no cholecystectomy group (358/1433 [25%]). (OR = 0.3; CI 0.17–0.51; *p* < 0.0001) (Fig. 8).

## Discussion

This meta-analysis identified that patients undergoing CBD clearance followed by prophylactic cholecystectomy had a significant reduction in biliary events, biliary pain and pancreatitis.

While patients in the community may be asymptomatic with silent CBD stones, those who present to hospital are often sick with potential sepsis, pancreatitis or obstructive jaundice. This is a disease process which is frequently fuelled by the persistent presence of cholecystolithiasis. The surgical community recognises that patients with asymptomatic cholelithiasis probably do not benefit from prophylactic cholecystectomy, but the current cohort of patients studied were symptomatic presenting with complicated CBD calculus disease.

There is significant variation in current practice in performing cholecystectomy in symptomatic patients with CBD stones and the definition of early cholecystectomy which, as reported by Nakai, can vary between studies from a mean of 8 days to up to 90 days [7]. Further, the reported cholecystectomy after ERCP for choledocholithiasis is only 22% and 8% in patients aged ≥ 75 years and ≥ 85 years, respectively [30]. A reason for this could be the comorbidity burden or frailty of the elderly patients. However, the age factor alone might also influence the surgical decision. With the increase in the elderly population worldwide and the concomitant CBD stones seen in patients with biliary calculus disease [33], an active approach is recommended even in this patient population if no absolute contraindication to anaesthesia or surgery exists. Ignoring the potential problems of CBD stones may be foolish as Hakuta and colleagues identified biliary complications, related to asymptomatic CBD stones picked up on incidental imaging, with a detection rate of 6.1% at 1 year, 11% at 3 years and 17% at 5 years [34]. Möller et al. found that among patients where no measures were taken intraoperatively or planned postoperatively, the possibility for adverse outcomes varied between 15.9 and 36.9% depending on stone size in a cohort of patients diagnosed with CBD stones using IOC [35].

There has been a paradigm shift in the management of acute biliary presentations in the last decade with increasing use of index or same admission cholecystectomy [36].

Concerns have been expressed about the risk of biliary injury with early surgery, but this remains unproven, overshadowed by the increasing concern about readmission and recurrent pancreatitis in the untreated cholecystitis patient [37]. Patients presenting with acute cholecystitis have a significant increased risk of CBD stones (10%), more than three times that seen in elective cholecystectomy. In these acute situations with sepsis, there is universal support for CBD clearance to facilitate sepsis control. What is more challenging is the decision of whether to remove the gallbladder or defer cholecystectomy in patients with CBD stones. While index admission cholecystectomy is increasingly advocated, it is not suitable for all patients and, in high-risk patients, it may be associated with adverse outcomes [37]. Mytton et al. suggest in their national cohort analysis in the UK that incentive to increase the number of index admission cholecystectomies may result in the risk of overtreating patients with cholecystitis [38]. While their study did not evaluate those who had CBD stones or clearance, one should always be cautious about the risk of overtreatment and a tailored risk assessment to prophylactic cholecystectomy should be undertaken [38]. A real-world clinical management challenge is to determine the relative contribution of cholecystitis versus cholestasis to obstructing CBD stones. Imaging may help predict the degree of cholecystitis, but it can be difficult to determine whether the dilatation/oedema is purely due to an obstructive rather than an infective element. As liver function tests become increasingly abnormal, there is a greater likelihood that the key process is CBD obstruction rather than cholecystitis. Failure to remove a septic gallbladder will increase mortality. Current preoperative grading systems offer some help, but have significant limitations [39]. Many scoring systems require surgery and thus are not useful in this situation [40].

Escourrou suggested, in 1984, that deferral of cholecystectomy in patients undergoing ERCP with endoscopic sphincterotomy (ES) was possible [41].

In a large study with a follow-up of 24.2 (< 1–82.3) months, Archibald found that cholecystectomy was eventually required in 46 (24.7%) of the deferred cholecystectomy (DC) patients on average 6 months after ES [22]. The younger subgroup underwent eventual cholecystectomy (57.6 v. 69.4 years; *p* < 0.001), had a lower ASA score (ASA score of 1.98 v. 2.26; *p* = 0.015) and had a higher chance of residual cholecystolithiasis than those with uneventful deferral. There was a higher occurrence of recurrent pancreatitis in the deferred cholecystectomy group (30%) compared to 4.8% in the prophylactic cholecystectomy [22]. Costi and colleagues, in their small study of octogenarian patients, referred to undergo LC post-ES and CBD clearance, found that while a wait-and-see policy allowed two-thirds to avoid surgery, biliary-related events developed for every second patient, often requiring surgery with eventual poorer outcomes, with 48% developing recurrent symptoms or complications [41]. A key to determining the need for post-ERCP cholecystectomy is the presence of residual cholelithiasis in the gallbladder, especially in patients with previous pancreatitis. In their retrospective study of over 160 patients post-CBD stone removal, Cui found that the incidence of acute cholecystitis was 13.6% compared to 2.5% in those without stones [24].

Physicians must weigh up the risks of complications if the gallbladder is left in situ against the risks associated with laparoscopic cholecystectomy after ERCP. Patients with ERCP clearance prior to cholecystectomy pose more technical operative difficulties leading to conversion rates of up to 20%, as reported by Lau et al. [28].

The amount of elderly patients presenting with gallstone disease is constantly rising and patients aged 80–89 years now account for 28% of male and 42% of female patients presenting with cholecystitis [43–45]. Elderly patients have increased comorbidities, reduced functional reserve and operating on elderly patients is associated with a higher risk of complications, higher conversion rates and a longer hospital stay [46]. In patients with acute cholecystitis, alternative strategies may include antibiotic management and percutaneous catheter drainage. However, a recent randomised controlled trial comparing percutaneous catheter drainage and laparoscopic cholecystectomy for high-risk patients with acute cholecystitis identified laparoscopic cholecystectomy to be associated with significantly lower rates of major complications and reinterventions [47].

Sousa, in a retrospective study of 131 patients (47 of whom were excluded for lack of data) with a mean age of 82 years, found a post-ERCP complication rate of 13% (8 pancreatitis, 6 cholangitis and 1 perforation) and a mortality rate of 0.7% in all patients. The same study found that 22% of patients had a cholecystectomy performed after ERCP with a median time to surgery of 209 days with a complication rate of 13% (1 empyema, 1 hemoperitoneum and 1 hypovolemic shock) and the mortality rate was 7%. Follow-up in this study revealed a new biliary event occurred in 20% of patients – 11% new ERCP, 9% cholecystitis, 9% cholangitis and 2% pancreatitis. Fewer biliary events were reported in patients who had a cholecystectomy (7% vs. 24%). No statistically significant difference in all-cause mortality (14% vs. 27%) or in mortality following biliary events (0% vs. 9%) [30]. However, it is important to highlight that in this meta-analysis the granular data on the cause and time of death was not readily available for analysis. Based on the risk of complications when deferring post-ERCP cholecystectomy, surgery should be offered to even elderly if no absolute contraindication to anaesthesia or surgery exists.

In their recent meta-analysis, McCarty found that patients with in situ gallbladders post-ES after ERCP for choledocholithiasis were found to have a 2.5-fold mortality rate and they highlighted that recurrent CBD stones occur in 37% of patients despite initial ERCP clearance [17]. Today, divergent views on the optimum CBD clearance technique exists. A recent meta-analysis by Ishii and colleagues, which compares BD and ES, identified that from collected data from the five RCTs and five NRCTs, the rate of complete CBD stone removal for the first session had a better outcome for endoscopic sphincterotomy followed by large balloon dilatation compared to endoscopic sphincterotomy (OR = 0.38, 95% CI 0.27–0.53, *p* < 0.01, *I*^2^ = 57%). They found that BD increased post-procedure pancreatitis but was also accompanied by less bleeding [48]

## Conclusion

Our meta-analysis, which includes both ES and BD to clear the CBD, found that prophylactic cholecystectomy resulted in a decreased risk of cholecystitis, cholangitis pancreatitis and mortality and would support prophylactic cholecystectomy following endoscopic clearance of CBD.
